# Quantitative Immunohistochemistry to Measure Regional Expression of Nurr1 in the Brain and the Effect of the Nurr1 Heterozygous Genotype

**DOI:** 10.3389/fnana.2021.563854

**Published:** 2021-04-30

**Authors:** Evangel Kummari, Shirley X. Guo-Ross, Heath S. Partington, Jennifer Makenzie Nutter, Jeffrey B. Eells

**Affiliations:** ^1^Department of Biology, Texas A&M University, College Station, TX, United States; ^2^Department of Basic Sciences, College of Veterinary Medicine, Mississippi State University, Starkville, MS, United States; ^3^Department of Anatomy and Cell Biology, Brody School of Medicine, East Carolina University, Greenville, NC, United States

**Keywords:** orphan nuclear receptor, immediate early gene, immunohistochemistry, dopamine, Nr4a2 expression

## Abstract

The transcription factor Nurr1 is a member of the steroid hormone nuclear receptor superfamily. Ablation of Nurr1 expression arrests mesencephalic dopamine neuron differentiation while attenuation of Nurr1 in the subiculum and hippocampus impairs learning and memory. Additionally, reduced Nurr1 expression has been reported in patients with Parkinson’s disease and Alzheimer’s disease. In order to better understand the overall function of Nurr1 in the brain, quantitative immunohistochemistry was used to measure cellular Nurr1 protein expression, across Nurr1 immunoreactive neuronal populations. Additionally, neuronal Nurr1 expression levels were compared between different brain regions in wild-type mice (+/+) and Nurr1 heterozygous mice (+/−). Regional Nurr1 protein was also investigated at various time points after a seizure induced by pentylenetetrazol (PTZ). Nurr1 protein is expressed in various regions throughout the brain, however, a wide range of Nurr1 expression levels were observed among various neuronal populations. Neurons in the parietal and temporal cortex (secondary somatosensory, insular, auditory, and temporal association cortex) had the highest relative Nurr1 expression (100%) followed closely by the claustrum/dorsal endopiriform cortex (85%) and then subiculum (76%). Lower Nurr1 protein levels were found in neurons in the substantia nigra pars compacta and ventral tegmental area (39%) followed by CA1 (25%) and CA3 (19%) of the hippocampus. Additionally, in the parietal and temporal cortex, two distinct populations of high and medium Nurr1 expressing neurons were observed. Comparisons between +/− and +/+ mice revealed Nurr1 protein was reduced in +/− mice by 27% in the parietal/temporal cortex, 49% in the claustrum/dorsal endopiriform cortex, 25% in the subiculum, 33% in substantia nigra pars compacta, 22% in ventral tegmental area, and 21% in CA1 region of the hippocampus. Based on these data, regional mechanisms appear to exist which can compensate for a loss of a Nurr1 allele. Following a single PTZ-induced seizure, Nurr1 protein in the dentate gyrus peaked around 2 h and returned to baseline by 8 h. Since altered Nurr1 expression has been implicated in neurologic disorders and Nurr1 agonists have showed protective effects, understanding regional protein expression of Nurr1, therefore, is necessary to understand how changes in Nurr1 expression can alter brain function.

## Introduction

The nuclear receptor Nurr1 (NR4A2) is structurally similar to the steroid-thyroid hormone nuclear receptors. Nurr1 can function as monomer or form dimers or a heterodimer with other nuclear receptors, such as Nur77, NOR-1, retinoid X receptor (RXR), or retinoic acid receptor (RAR), to bind to DNA response elements, such as the Nur binding response element (NBRE) and Nur response elements (NurRE), to regulate target gene expression (Wallen-Mackenzie et al., 2003). The distribution of Nurr1 mRNA expression in the brain has been previously reported (Reviewed in Eells et al., 2000). Based mostly on *in situ* hybridization and some immunohistochemistry studies, the distribution of Nurr1 in the brain includes dopamine neurons in the substantia nigra pars compacta (SNpc) and ventral tegmental area (VTA) (Backman et al., 1999), glutamatergic neurons in the claustrum (Watakabe et al., 2014), dorsal endopiriform cortex, subiculum (Moon et al., 2015, 2019), and in the deep layer (VIb) of frontal cortex (Arimatsu et al., 2003). Within the frontal cortex Nurr1 is a marker for cortical sub-plate neurons in layer VIb and co-localizes with Ca^2+^/calmodulin kinase IV expression (Arimatsu et al., 2003; Navarro et al., 2014). Expression was also located in scattered neurons in the insular and secondary somatosensory cortex (Arimatsu et al., 2003) and hippocampus (Saucedo-Cardenas and Conneely, 1996).

The first functional role discovered for Nurr1 was the necessity of Nurr1 for terminal differentiation of midbrain dopamine neurons (Saucedo-Cardenas and Conneely, 1996; Zetterstrom et al., 1997; Castillo et al., 1998). Subsequent studies have found a role in maintaining the dopamine phenotype in adults as well as regulating expression of dopamine neurotransmission genes and ultimately dopamine neurotransmission (Eells et al., 2002, 2006; Kadkhodaei et al., 2009, 2013). Nurr1 in dopamine neurons has also been implicated in the regulation of some mitochondrial genes which could provide a clue for the role of Nurr1 in other neurons (Kadkhodaei et al., 2013). In addition to Nurr1 function in dopamine neurons, a role for Nurr1 has also been demonstrate in the hippocampus. Attenuating Nurr1 expression in the hippocampus was reported to diminish performance on spatial long term memory (Colon-Cesario et al., 2006). More recently, attenuating Nurr1 expression in the subiculum enhanced neuropathology in an Alzheimer’s disease model while elevating Nurr1 expression attenuated the neuropathology (Moon et al., 2019). The Nurr1 agonist amodiaquine also reduced neuropathology and was associated with improvements in learning and memory (Moon et al., 2019).

Another important aspect to Nurr1 function is the regulation of its expression levels. Altered expression and function of Nurr1 has also been implicated in several diseases, including addiction, schizophrenia, Parkinson’s disease, and Alzheimer’s disease. Patients with substance use disorders (i.e., cocaine and morphine addiction) were shown to have altered Nurr1 expression within the dopaminergic system (Bannon et al., 2002, 2004; Koo et al., 2015). Schizophrenia patients had also been reported to have reduced Nurr1 expression in the cortex (Xing et al., 2006; Corley et al., 2016). Within the dopamine neurons, Nurr1 levels were reduced with aging and in Parkinson’s disease (Chu et al., 2002, 2006). More recently, a reduction in Nurr1 expression in the cortex and subiculum was associated with learning impairments and neuropathology in a mouse model of Alzheimer’s disease as well as correlated with neuropathology in tissue from patients with Alzheimer’s disease (Moon et al., 2015, 2019). Based on these data, regional changes in Nurr1 protein expression could have an important role in neuropathological disease.

Because of the widespread distribution of Nurr1 and the importance of Nurr1 expression levels with regard to function, the current study investigated the cellular expression levels of Nurr1 protein across various brain regions. Although the distribution of Nurr1 mRNA and protein has been described, the relative expression levels of Nurr1 across various neuronal populations have not been reported. Popular protein quantification methods like western blot and ELISA are not yet sensitive enough for quantification in an individual neuron. Here we use a quantitative immunohistochemistry (Q-IHC) approach to identify Nurr1 protein expressing cells as well as get a quantitative value for Nurr1 expression. The main advantage of this method is that we can identify and quantify nuclear Nurr1 protein of individual cells. Potential observer bias is greatly reduced, as compared to a qualitative assessment, when specific cell regions are being examined with Q-IHC.

In this study, a global assessment of Nurr1 protein expression throughout the brain in mice is described and compared to published data on Nurr1 mRNA expression. Additionally, we used Q-IHC to study the Nurr1 protein levels across populations of Nurr1 expressing neurons in subiculum, parietal and temporal cortex, the deep temporal nuclei which included the claustrum and dorsal endopiriform nucleus, CA1 and CA3 areas of the hippocampus, SNpc, and VTA. Our results demonstrate a wide range of Nurr1 expression levels across regions, with lowest expression in the SNpc/VTA and the hippocampus. Additionally, reductions in Nurr1 protein expression in the Nurr1 +/− genotype across regions was not uniform, with the greatest reduction in the parietal/temporal cortex. Additionally, following a single pentylenetetrazol (PTZ)-induced seizure, we demonstrate a peak in Nurr1 protein expression in the dentate gyrus at around 2 h and a return to baseline by 8 h. These data are significant because they provide a better understanding of Nurr1 function in the brain and important insight into how alterations in Nurr1 expression and/or function could affect the brain.

## Materials and Methods

### Experimental Animals

The Nurr1 +/− and +/+ mice used for this study were from a colony bred at Mississippi State University and East Carolina University. They were maintained at 18–22°C with food and water *ad libitum*. They were weaned at the age of 19–21 days and housed 3–5 per cage. Genotyping was done using tail DNA to differentiate between +/− and +/+ as described previously by Castillo et al. (1998). All procedures performed on these mice were in accordance with the National Institutes of Health Guide for the Care and Use of Laboratory Animals, and the study protocols were approved by the Institutional Animal Care and Use Committee at Mississippi State University and East Carolina University. The animals used in this project were housed in the AAALAC accredited facilities of the College of Veterinary Medicine, Mississippi State University and Brody School of Medicine, East Carolina University. These studies investigated Nurr1 immunohistochemistry in +/+ and Nurr1 +/− mice. Additionally, in wild-type mice, Nurr1 protein distribution was examined after either a vehicle injection or at different times after a clonic-tonic seizure induced with 50 mg/kg PTZ. All PTZ injected mice experienced a stage 6 clonic, tonic-clonic seizure based on the revised Racine scale with rearing and falling, facial and forelimb clonus followed by tonic forelimb extension as described by Van Erum on using PTZ seizures in mice (Van Erum et al., 2019). The mice were euthanized with CO_2_ asphyxiation and decapitated. The brains were removed, frozen on dry ice, and stored at −80°C. For global Nurr1 expression on free floating sections, brains were emersion fixed in 4% paraformaldehyde for either 24 h, cryoprotected in 30% sucrose, sectioned on a cryostat and processed for free floating immunohistochemistry. Paraformaldehyde fixed brains were serially sectioned at 30 μm thickness and collected in PBS as free floating sections. Fresh frozen brains from +/+ and +/− mice were serially sectioned on a Microm HM 560 cryostat into 10 μm sections onto silanized slides. Nurr1 +/− mice were breed together and embryos at approximately embryonic day 14 were obtained. Tissue from embryos were genotyped to determine embryos homozygous for the Nurr1 knockout allele. Male mice aged 90–120 days of age were used in these studies. For Nurr1 distribution studies, sections from four mice were assessed. Serial fresh frozen section from 1 +/+ mouse was used for antibody concentration measurements. Three wild-type and three heterozygous mice were used to quantify Nurr1 protein expression across regions and genotype. For PTZ treatments one mouse was used at each time point.

### Nurr1 Immunohistochemistry – Free Floating Sections

In preliminary studies using this antibody, we found that antibody recognition was very sensitive to fixation. In fresh frozen section, 1 h of fixation is both necessary and sufficient for antibody signal. Longer fixation diminished the immunohistochemistry signal. Whole brain tissue emersion fixed in 4% paraformaldehyde for 24 h, however, required antigen retrieval. For antigen retrieval, these sections (30 mm) were incubated in 10 mM citrate buffer for 20 min at 95°C and then washed in PBS three times. Sections were incubated with 1% H_2_O_2_ in PBS for 30 min then washed in PBS three times. Section were then blocked with 10% rabbit normal serum in 1% BSA-0.1% Triton-X100-PBS for 45 min. After blocking the sections were incubated with goat anti-Nurr1 primary antibody (R&D Systems AF2156) diluted to a concentration of 10 nM (1:133 from stock of 200 mg/ml) in PBS-BSA-Triton overnight at 4°C. Sections were then washed three times in PBS and incubated with biotinylated rabbit anti-goat (1:200) secondary antibody in PBS-BSA-Triton for 2 h. The sections were then washed three times in PBS and incubated with ABC reagent (made 30 min before to equilibrate) for 2 h followed by three washes in PBS. Sections were incubating for 5–10 min in 0.5X of 10 × 3,3′-Diaminobenzidine (DAB) Liquid Substrate in PBS with 0.003% hydrogen peroxide (Sigma-Aldrich) or in Tyramide-488 or 594 in incubation buffer (Biotium). Sections were rinsed in PBS for three times, mounted on silane prep slides and air dried at room temperature for 30 min. DAB labeled sections were then dehydrated in graded ethanol, cover-slipped using Permount and air dried overnight at room temperature. Tyramide labeled sections were mounted on slides, labeled with DAPI then coverslipped with EverBrite^TM^ Hardset mounting medium (Biotium). For duel Nurr1-tyrosine hydroxylase immunohistochemistry, following Nurr1 immunohistochemistry, sections were washed three times and placed into a 4% goat serum (Vector S-1000, Burlingame, CA, United States) in PBS-1% BSA-0.1% TritonX-100 for 1 h. Sections were then incubated in a rabbit anti-TH primary antibody (Millipore AB152, Darmstadt, Germany) diluted 1:1000 in PBS-1% BSA-0.1% TritonX-100 overnight at 4°C. Sections were then washed five times in PBS-0.1% BSA and placed into goat-Anti-Rabbit IgG secondary antibody conjugated to AlexaFluor594 (Invitrogen A11008, Rockford, IL, United States) diluted 1:500 in PBS-1% BSA-0.1% TritonX-100 for 2 h. Sections then underwent five PBS washes, mounted on silanized slides, DAPI stained, and coverslipped using EverBrite (Biotium 23003, Fremont, CA, United States) mounting media. Photomicrographs were taken using the using Olympus BX51 microscope or the Celldiscoverer 7. These sections were used to demonstrate regional expression of Nurr1.

### Nurr1 Immunohistochemistry – Fresh Frozen Section

Fresh frozen section mounted on silanized slides were fixed in 4% paraformaldehyde for 1 h at room temperature, washed three times in phosphate buffered saline (PBS), and then incubated with 1%H_2_O_2_ in PBS for 30 min. Sections were washed in PBS three times followed by blocking in 10% rabbit serum in PBS-BSA-Triton X100 for 45 min. Sections were then dipped in PBS once and incubated overnight at 4°C with a goat anti- Nurr1 primary antibody (R&D Systems AF2156) diluted in PBS-BSA-Triton at the following varying concentrations: 3 nM (1:444), 10 nM (1:133), 20 nM (1:66.5), 30 nM (1:44), or 60 nM (1:22). Sections were then washed five times in PBS containing 0.1% BSA, rinsed once in PBS and incubated for 2 h with biotinylated rabbit anti-goat secondary antibody (Vector Laboratories) at a dilution of 1:200 in PBS-BSA-Triton. This was followed by washing three times in PBS and incubating with ABC reagent (Vector Laboratories) for 2 h, then followed by three washes in PBS. Tissue was then incubated for 5 min in 0.5X of 10 × 3,3′-Diaminobenzidine (DAB) Liquid Substrate in PBS with 0.003% hydrogen peroxide (Sigma-Aldrich). Sections were then washed in PBS, dehydrated in graded ethanol followed by xylene and coverslipped using Permount. A concentration of 20 nM Nurr1 primary antibody was used to compare regional Nurr1 expression in Nurr1 +/− and +/− mice. The specificity of Nurr1 primary antibody was tested using brain tissue from an approximately 14 day old embryo lacking the Nurr1 gene and compared to a wild-type embryo.

### Quantitative Immunohistochemistry

Nurr1 immunohistochemistry on fresh frozen sections was used in this experiment to measure the amount of Nurr1 protein expression in six different regions of the brain, comparing +/+ and +/− mice, and after a PTZ seizure. The six different regions were the subiculum, parietal/temporal cortex, the claustrum/dorsal endopiriform cortex, CA1 area of the hippocampus, SNpc, and VTA. Different concentrations of Nurr1 antibody were also tested to ensure that Nurr1 expressing cells were adequately stained in regions of low and high expression. The different concentrations used were 3 nM, 10 nM, 30 nM, and 60 nM. Based on this data, the concentration of 20 nM was then used to further study the intensity of Nurr1 expression in frozen sections.

Photomicrographs of these six Nurr1 expressing regions were obtained using Olympus BX51 microscope, which has a fluorescent lamp and fluorescent filters with a CCD camera and a motorized Z-stage, connected to a computer with Stereo Investigator Stereology Software from MicroBrightField Inc. Using Stereo Investigator software, the height and width of the digital image frame were set at 268.5 mm and 174.0 mm, respectively, for the 40x field. Each individual region was outlined at 10x magnification. Stereo Investigator software was used to obtain unbiased fields and digital images were acquired at 40x magnification with an oil immersion objective. For each region, 3–5 sections were samples with 4–6 frames captures and approximately 40–80 nuclei were measured (see Figure 2).

After procuring digital images, each Jpeg image obtained was opened in Photoshop (Adobe, San Jose, CA, United States). The marquee tool was used to select a 30 × 30 pixel square in the nucleus of neurons in the images (Figure 3). The Nurr1 immunoreactive nuclei were identified and cut out using the marquee tool, then pasted and stored in a new file in TIFF format. A region of no chromogen stain was also identified and used as a background control. This background intensity was used to standardize measurements across samples and procedures.

Matlab software was used to determine the pixel energy of the labeled nuclei using the program described by Matkowskyj et al. (2000). All data was reported as mean and SEM.

## Results

### Regional Nurr1 Protein Expression

**FIGURE 1:**
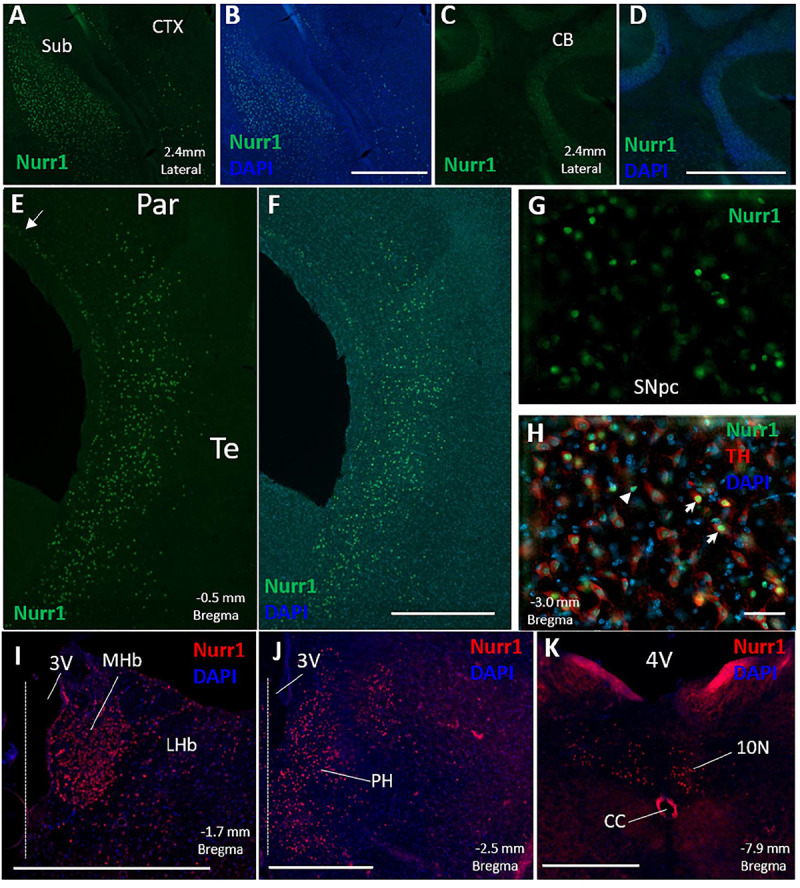
Distribution of Nurr1 protein expression is shown in the mouse brain based on Nurr1 immunofluorescence (IF) in free floating sections. Nurr1 expression was observed in a dense population of cells in the subiculum (**A**-Nurr1 IF 488, **B**-Nurr1 IF 488 + DAPI). No discernable Nurr1 IF was observed in the cerebellar cortex (**C**-Nurr1 IF 488, **D**-Nurr1 IF + DAPI). Intense Nurr1 immunofluorescence was found in neurons scattered throughout layers IV–VI of the parietal and temporal cortex consisting of secondary somatosensory cortex, insular cortex, auditory cortex, and temporal association cortex **(E,F)**. Near the boundary between secondary and primary somatosensory cortex, Nurr1 immunoreactive neurons were located in the deep layer (arrow) of the primary somatosensory cortex extending into motor cortex medially (**E**-Nurr1 IF 488, **F**-Nurr1 IF 488 + DAPI). Nurr1 IF was also observed in the substantia nigra pars compacta and ventral tegmental area where Nurr1 co-localized with tyrosine hydroxylase (**G**-Nurr1 IF 488, **H**-Nurr1 IF 488 + Tyrosine hydroxylase IF 594 + DAPI). Although most tyrosine hydroxylase immunofluorescent neurons co-labeled with Nurr1 (white arrows), a population of Nurr1 immunofluorescent cells did not have tyrosine hydroxylase (white arrowhead) **(H)**. Nurr1 immunoreactive neurons, labeled with tyramide 594, were also observed in the medial and lateral habenula **(I)**, posterior hypothalamus **(J)**, and dorsal motor nucleus of the vagus **(K)**. Dashed line in **(I,J)** represent the location of the midline. The location of brain structures (lateral from midline and distance from bregma) labeled in these figures **(A–K)** is based on the stereotaxic maps from the mouse brain atlas of Paxinos and Franklin (2019). CB, cerebellar cortex; CC, central canal; CTX, cerebral cortex; LHb, lateral habenula; MHb, medial habenula; Par, parietal cortex; Sub, subiculum; SNpc, substantia nigra pars compacta; Te, temporal cortex; 3V, 3rd ventricle; 4V, 4th ventricle. Scale Bar = 500 μm **(A–F,I–K)** and 50 μm **(G,H)**.

**FIGURE 2:**
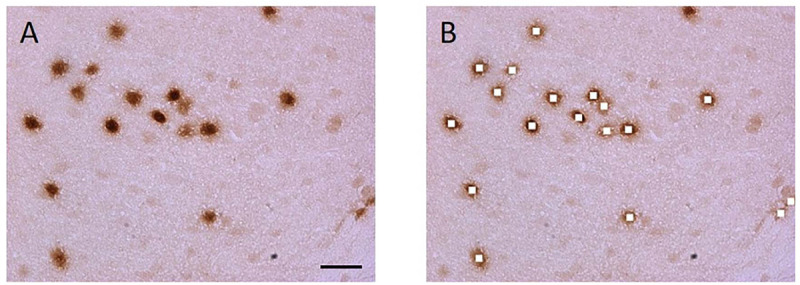
Nurr1 labeled nuclei were cut out and the pixel energy quantified using Matlab software. Unbiased microscope fields were obtained with Stereo investigator software. **(A)** Section containing Nurr1 labeled DAB stained nuclei. **(B)** The stained nuclei were cut using Adobe Photoshop software. Scale bar = 20 μm.

Based on immunofluorescence for Nurr1 on free floating sections, the densest population of Nurr1 protein expressing cells was found in the claustrum, dorsal endopiriform cortex, and the subiculum (Figures 1A,B, 2). Additionally, Nurr1 immunofluorescence was found in cells scattered throughout layers IV–VI of the parietal and temporal cortex. This expression pattern was closely associated with secondary somatosensory cortex rostrally, beginning around 0.4 mm rostral to bregma, and continuous with auditory cortex, temporal association cortex, lateral secondary visual, and insular cortex. We subsequently refer to this cortical expression collectively as parietal/temporal cortex for simplicity. Dorsally and medially, this patterned of scattered labeling appeared to stop mostly at the boundary between secondary and primary somatosensory cortex. At this point, Nurr1 immunofluorescent cells were observed only in the deep layer of the primary somatosensory and motor cortex medially (Figures 1E,F). Additionally, Nurr1 expression was observed in the SNpc and VTA, as has been previously reported. In double labeling with Nurr1 and tyrosine hydroxylase immunohistochemistry, most tyrosine hydroxylase immunoreactive neurons were also immunoreactive for Nurr1. However, a small number of Nurr1 immunoreactive nuclei (approximately 1–2% of all immunofluorescent nuclei in the SNpc and VTA) were not immunoreactive for tyrosine hydroxylase (Figures 1G,H). Light labeling was observed in CA1/CA3 region of the hippocampus. Other regions with Nurr1 protein expression included medial and lateral habenula, posterior hypothalamus, and the motor nucleus of the vagus nerve (Figures 1I–K). One area where Nurr1 protein expression was absent was the cerebellar cortex (Figures 1C,D). This is of interest as other publications using *in situ* hybridization reported strong Nurr1 mRNA expression.

### Titration of Nurr1 Antibody for Nurr1 Expression

**FIGURE 3:**
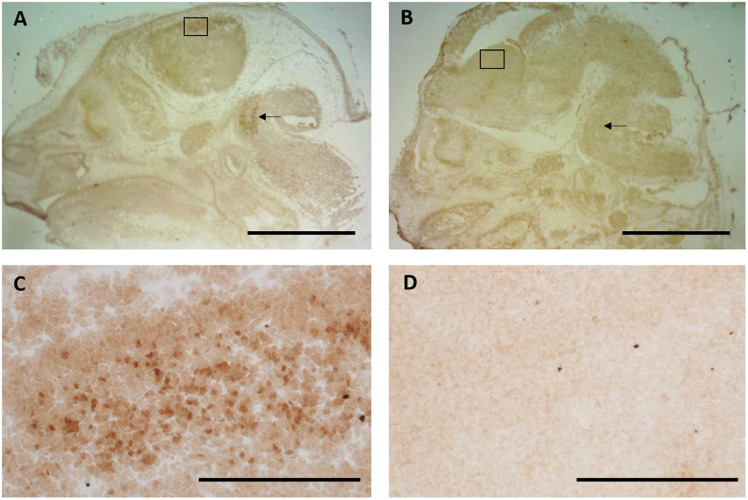
Specificity of the Nurr1 antibody was tested using tissue from a Nurr1 knockout 14 days embryo. Clear specific labeling was observed in the cortex (**A**-box and **C**-high magnification of box) and brainstem (arrow) in wild-type tissue that was absent in the Nurr1 knockout **(B,D)**. This data clearly shows this Nurr1 antibody to be specific for the Nurr1 protein. **(A,B)**: Scale bar = 2 mm, **(C,D)**: Scale bar = 100 μm.

**FIGURE 4:**
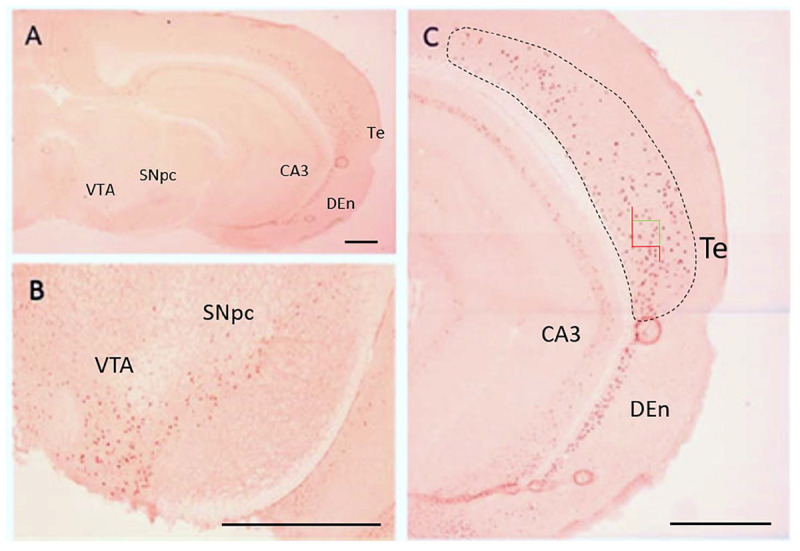
Distribution of Nurr1 protein expression in the brain based on Nurr1 immunohistochemistry in frozen sections for Q-IHC **(A)**. Nurr1 expression is shown in SNpc and VTA region **(B)**, temporal cortex, CA3 of the hippocampus, and dorsal endopiriform cortex **(C)**. A representation of the procedure used to acquire images is show for the temporal cortex in which the region of interest is outlined (dashed line) and Stereo Investigator software used to acquire images at 40X magnification (green and red box). DEn, dorsal endopiriform cortex; SNpc, substantia nigra pars compacta; Te, temporal cortex; VTA, ventral tegmental area. Scale Bar = 1000 μm.

**FIGURE 5:**
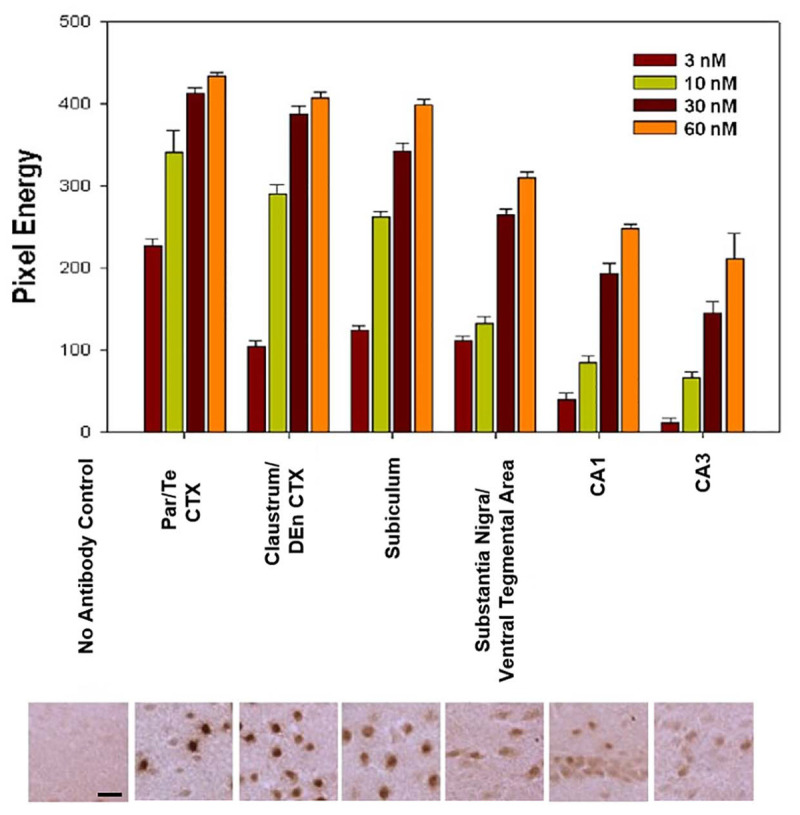
Nurr1 immunoreactivity was quantified in different regions and at different primary antibody concentrations. Differences in Nurr1 expression are shown across different regions. Based on chromogen intensity using a primary antibody concentration of 10 nM, neurons in the parietal/temporal (Par/Te) cortex had the highest relative Nurr1 expression (100%) followed closely by the claustrum/dorsal endopiriform cortex (85%) and subiculum (76%). Lower Nurr1 levels were found in the substantia nigra and ventral tegmental area (39%) followed by CA1 region (25%) and CA3 region (19%) of the hippocampus. Pixel energy is the average and SEM for all nuclei measured. Photomicrographs below the graph are representative 40X magnification images acquired within each above area for quantification. Scale bar = 20 mm.

For Q-IHC, 10 mm fresh frozen sections processed for Nurr1 immunohistochemistry were used because it provided very good labeling with low background. Using brain tissue for a Nurr1 knockout embryo we tested the specificity of the Nurr1 antibody. Specific labeling was observed in the Nurr1 wild-type tissue in the cortex and brainstem that was clearly absent in the knockout tissue (Figure 3). This data show this Nurr1 primary antibody is specific for Nurr1. Based on the studies investigating the distribution of Nurr1 protein, there appeared to be a wide range of immunoreactive intensities for Nurr1 amongst the identified regions (Figure 3). For each area quantified, Stereo Investigator software was used to outline the region of interest and randomly acquire images. A representation of this process is shown for the parietal/temporal cortex in Figure 3C. Individual nuclei were cut out and intensity measured (Figure 4). In order to investigate this difference across regions, Nurr1 immunoreactivity was quantified in selected regions using a range of primary antibody concentrations of 3 nM, 10 nM, 30 nM, and 60 nM (Figure 2). Using different antibody concentrations, the range of Nurr1 immunoreactivity was evident (Figure 5). When the intensity of Nurr1 immunoreactivity was quantified, the Nurr1 immunoreactive nuclei in the parietal/temporal cortex were the most darkly stained followed by claustrum/dorsal endopiriform cortex and subiculum (Figure 2). The neurons in the SNpc and VTA showed considerably less intensity followed by neurons in CA1 and CA3 regions of the hippocampus. In sections stained with 3 nM and 10 nM of Nurr1 antibody, Nurr1 immunoreactivity was evident in the areas with high Nurr1 expression but was undetectable from areas with low Nurr1 expression. A 60 nM antibody concentration resulted in very dark labeling in all regions. In high expressing areas, the density of labeling was no longer different between these areas. Whereas, labeling using the 3 nM antibody concentration showed higher densities in the parietal/temporal cortex as compared to claustrum/dorsal endopiriform cortex and subiculum, similar densities in these areas were obtained from the 30 nM concentration. Primary antibody concentrations of 10 and 30 nM gave the best distribution of Nurr1 intensity. Therefore, a primary antibody concentration of 20 nM was chosen for subsequent studies comparing +/+ and +/− mice. These studies demonstrate both the distribution of Nurr1 protein expression but also the magnitude of Nurr1 expression.

### Wide Range of Nurr1 Protein Expression Across Various Nuclei

**FIGURE 6:**
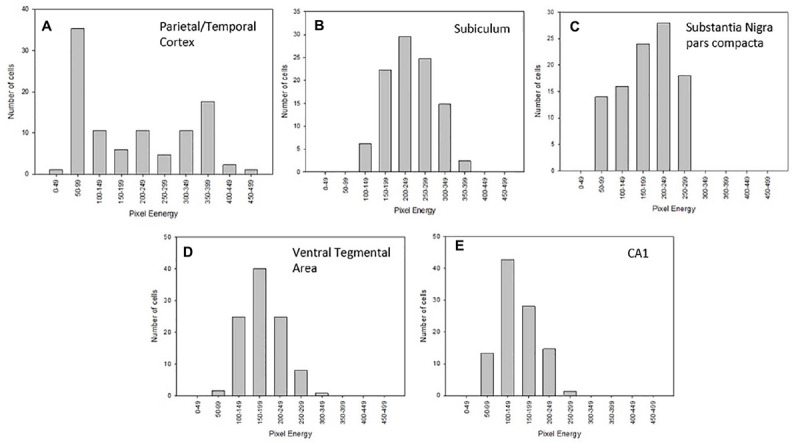
Histogram analysis of Nurr1 protein expression in the parietal/temporal cortex **(A)**, subiculum **(B)**, substantia nigra-pars compacta (SNpc-**C**), ventral tegmental area (VTA-**D**), and CA1 of the hippocampus **(E)** using data associated with 10 nM Nurr1 primary antibody concentration on fresh frozen section. The Nurr1 labeled nuclei were analyzed using the highest 50 cells with increasing intensity. Within the parietal/temporal cortex, a wide distribution of intensities was observed, with peaks at low and high values. The analysis of the other areas revealed a more homogeneous distribution. Analysis is based on measurements from three mice.

Considerable regional differences in Nurr1 protein expression was observed based on intensity of staining. Based on chromogen intensity, at a 10 nM antibody concentration, neurons in the parietal/temporal cortex had the highest relative Nurr1 expression (100%) followed closely by the, claustrum/dorsal endopiriform cortex (85%), and subicullum (76%). Lower Nurr1 levels were found in the dopamine neurons in the SNpc and VTA (39%) followed by CA1 region (25%) and CA3 region (19%) of the hippocampus. In the subiculum, claustrum/dorsal endopiriform cortex, CA regions of the hippocampus, SNpc, and the intensity of immunoreactivity was more uniform with a narrow range of intensities (Figures 6B–E). In contrast, within the parietal/temporal cortex, when the intensity of immunostaining was plotted as a histogram, two different populations of Nurr1 expressing nuclei were observed based on Nurr1 intensity. The neurons in the parietal/temporal cortex had high Nurr1 expressing nuclei as well as low to medium expressing population of nuclei based on the intensity of labeling (Figure 6A).

### Regional Variation in Reduction of Nurr1 Expression in Nurr1 +/− Mice

**FIGURE 7:**
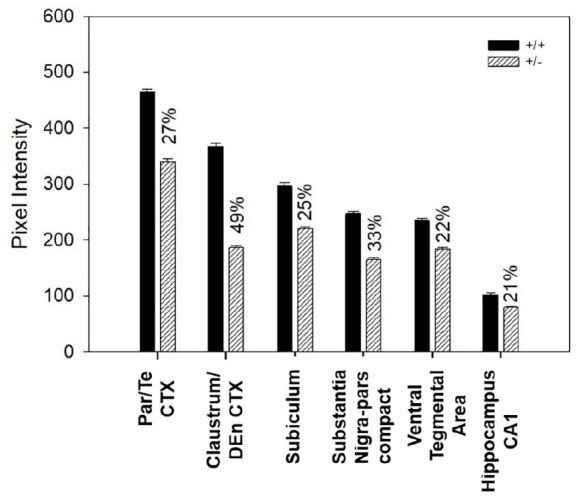
Difference in Nurr1 protein expression between heterozygous (*n* = 3) and wild type Nurr1 mice (*n* = 3) in six different regions of the brain using 20 nM of Nurr1 primary antibody concentration. Based on chromogen intensity, neurons of the +/− mice had significantly lower Nurr1 expression as compared to the +/+ mice but this varied across region. Magnitude of reductions in the +/− mice were 27% for the parietal/temporal cortex, 49% for the claustrum/dorsal endopiriform cortex, 25% for subiculum, 33% for substantia nigra pars compacta, 22% for ventral tegmental area, and 21% for CA1 region of the hippocampus.

Since difference in regional intensity of Nurr1 immunoreactivity was observed, the next set of experiments investigated intensity of Nurr1 immunoreactivity across brain regions in Nurr1 +/− mice. Nurr1 nuclei in +/− mice, as compared to +/+ mice, had reduced Nurr1 protein expression. Based on chromogen intensity calculated using Matlab and compared to the average intensity in wild-type mice, Nurr1 immunoreactive nuclei of the parietal/temporal cortex in +/− mice had a 27% reduction of Nurr1 protein. In the claustrum/dorsal endopiriform cortex, 49% less Nurr1 protein was found in +/− mice as compared to the +/+ mice. There was 25% less Nurr1 in subiculum, 33% less in SNpc and 22% less in VTA, and 21% less in CA1 region of the hippocampus in +/− than in +/+ Nurr1 mice (Figure 7).

### Time Course of Nurr1 Protein Expression After a Tonic-Clonic Seizure

**FIGURE 8:**
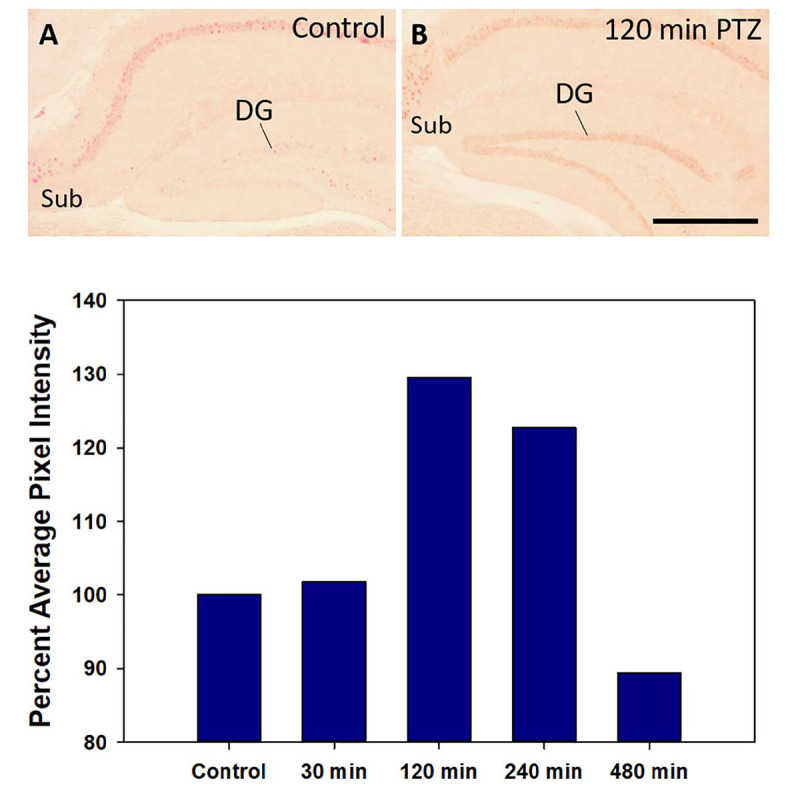
Nurr1 protein expression in the hippocampus in a control mouse **(A)** and 120 min after a PTZ seizure **(B)**. Relative pixel energy for Nurr1 immunohistochemistry in the dentate gyrus (DG) of the control (100%) was compare to pixel energy in the DG 30 min, 120 min, 240 min, and 480 min after a single PTZ seizure. Quantification of Nurr1 protein expression based on relative pixel energy across time after a single PTZ demonstrates at peak around 120 min that returns to near basal level by 480 min. Scale bar = 200 μm.

In preliminary studies, Nurr1 protein expression was examined at various times after a PTZ-induced seizure, and compared to a vehicle-injected control, using one mouse at each time point. In all mice injected with PTZ, a seizure consisting of facial and forelimb clonus followed rapidly by generalized tonic forelimb extension was induced. Mice were euthanized 30 min, 120 min, 240 min, and 480 min following the seizure. A mouse that was injected with saline and did not receive PTZ was used as control. Based on Nurr1 immunohistochemistry, changes in the distribution of Nurr1 protein was not noticeably alter by PTZ seizure with the exception of the dentate gyrus of the hippocampus (Figures 8A,B). Therefore, the intensity of Nurr1 immunoreactivity in the dentate gyrus was quantified at these different time points. Nurr1 immunoreactivity was not increased by 30 min after seizure. By 120 min after the seizure, Nurr1 immunoreactivity increased by 30% and this remained elevated at 240 min (22% increase). By 480 min after the seizure, Nurr1 immunoreactivity had returned close to base line. The data demonstrate, at least for the dentate gyrus, that new Nurr1 protein requires between 1 and 2 h for expression and duration of expression is between 4 and 8 h.

## Discussion

The current study investigated the distribution and quantification of Nurr1 protein in six different regions of the brain, namely parietal/temporal cortex (consisting primarily of secondary somatosensory cortex, temporal association cortex, auditory cortex, and insular cortex), subiculum, claustrum/dorsal endopiriform cortex, CA1/CA3 regions of the hippocampus, and SNpc/VTA using Q-IHC. In contrast to other immunoassays like Western blot and ELISA that determine total region protein levels, Q-IHC enables protein measurements in individual neurons. Here we used Q-IHC as a method to measure expression of Nurr1 protein in neurons across different anatomical locations, in a manner similar to quantification of RNA with *in situ* hybridization. To our knowledge, however, an evaluation of Nurr1 protein expression throughout the brain has not be previously reported. Using this specific method, along with sampling regions in an unbiased manor, we were able to quantify the relative amount of Nurr1 protein in individual neurons as an average across these neuronal populations.

Several published studies, including images available on the Allen Brain Atlas, have used *in situ* hybridization data to demonstrate the distribution of Nurr1 mRNA expression throughout the brain (Saucedo-Cardenas and Conneely, 1996; Xiao et al., 1996; Xing et al., 1997). Although some have high background, most reports show a similar distribution pattern to the IHC reported here. We did find one notable exception. Previous *in situ* hybridization data showed a strong signal for Nurr1 mRNA in the cerebellar cortex (Saucedo-Cardenas and Conneely, 1996; Xiao et al., 1996; Xing et al., 1997). However, no detectable Nurr1 IHC signal was observed in the cerebellar cortex. Using our IHC data, specific Nurr1-immunoreactive neurons are more readily differentiated from the background labeling. Additionally, we demonstrate the specificity of the Nurr1 antibody used based on a similar expression pattern in the brain of wild-type embryos and the absence of this signal in the brain of a Nurr1 knockout embryo. Therefore, this characteristic distribution pattern for Nurr1 protein expression can serve as a useful control to verify specific Nurr1 immunoreactivity.

Here we confirm the expression pattern of Nurr1 protein but also, based on our data using different concentrations of Nurr1 antibody, we were able to demonstrate a wide range of Nurr1 expression levels. In contrast, a previous publication using a qualitative assessment of expression level, reported the same moderate expression across the cortex, subiculum, hippocampus and SNpc/VTA (Luo et al., 2009). Our data, however, found lower levels of Nurr1 expression in neurons in the CA1/CA3 region of the hippocampus and SNpc/VTA as compared to the parietal/temporal cortex and subiculum. Both the number of neurons expressing Nurr1 as well as the amount of Nurr1 in each neuron appears to be important for function. Areas of the brain where a function for Nurr1 has been reported include the dopamine neurons in the SNpc and VTA and in the hippocampus, specifically CA1/CA3. Interestingly, these two areas express a relatively low level of Nurr1 protein. Within the dopamine neurons, Nurr1 is required for the development and maintenance of the dopaminergic phenotype and expression of dopamine neurotransmission genes (Eells et al., 2002, 2006; Kadkhodaei et al., 2009, 2013). Within the hippocampus, Nurr1 expression is elevated with certain memory tasks and blocking this induction of Nurr1 expression impairs memory (Pena de Ortiz et al., 2000; Colon-Cesario et al., 2006). Currently, a functional role for Nurr1 in the higher expressing regions has not been reported.

Although little is known about the function of Nurr1 in areas outside of the mesencephalic dopamine neurons and in the hippocampus, some anatomical data about these Nurr1 immunoreactive neuron populations have been reported. The claustrum is located in the deep temporal/insular cortex region. Functionally, the claustrum is thought to coordinate activation across cortical areas as these neurons received and project back to multiple cortical regions (Dillingham et al., 2017). Within the claustrum, Nurr1 labels most of the neurons which have been identify as glutamatergic (Fremeau et al., 2001; Hur and Zaborszky, 2005). Based on Nurr1 IHC, these neurons are similar in origin to the Nurr1 expressing neurons in the insular cortex (Watakabe, 2017). Within the subiculum, another area with relatively high Nurr1 expression, Nurr1 is also expressed in all neurons of this region which are also glutamatergic, based on co-expression with excitatory amino acid transporter (Moon et al., 2015). The subiculum receives afferent input from neurons in CA regions and shares efferent targets with these neurons as well as having some exclusive efferent projects such as to the anterior thalamus and mammillary bodies [Reviewed in Aggleton and Christiansen (2015)]. The subiculum appears to be involved, along with the hippocampus, in spatial memory tasks. However, more recent data from Roy et al. suggests more involvement in memory retrieval (Roy et al., 2017). Although a function for Nurr1 in the subiculum neurons has not been reported, both aging and the number of amyloid immunoreactive neurons in the 5XFAD mouse model of Alzheimer’s disease, decreased the number of Nurr1 expressing neurons in the subiculum (Moon et al., 2015). These alterations in Nurr1 expression were also associated with a reduction in fear conditioning (Moon et al., 2015). Additionally, b-amyloid accumulation in 5XFAD mice was exacerbated by attenuation of Nurr1 expression, via sh-RNA, but diminished by over expression of Nurr1 (Moon et al., 2019). Treatment with the highly selective Nurr1 agonist amodiaquine reduced Aβ plaques and improved cognitive function (Moon et al., 2019). Although this data does not determine the mechanism of Nurr1 action in the subiculum, it does demonstrate an important role for alterations in Nurr1 expression in regulating overall function.

The neurons with the highest expression levels of Nurr1 protein were located in the parietal and temporal cortex, which included secondary somatosensory cortex, insular cortex, auditory cortex, and temporal association cortex. In addition to this high Nurr1 expressing population, a separate populations of dopamine neurons in this region were observed with a lower level of Nurr1 expression. Based on available data, Nurr1 in the parietal/temporal cortex appears to be expressed in glutamate neurons. It remains to be determined if the high and medium Nurr1 expressing neurons represent different anatomical or functional populations of cortical neurons. To better characterize the distribution of Nurr1 expression in the brain, future studies will focus on identifying these specific populations of neurons in the temporal and parietal cortex. Additionally, Nurr1 immunoreactive neurons were observed in the deep layers of frontal and parietal cortex. In the deep layers of the rest of the cortex, Nurr1 has been shown to be a marker for neurons in layer VIb (Arimatsu et al., 2003). Currently, the function of Nurr1 in these neurons has not been determined.

Previous studies have reported reduced Nurr1 protein expression in the Nurr1 +/− mice (Luo et al., 2010; Rojas et al., 2010). Using the Q-IHC technique, it was possible to determine if fewer neurons were making the protein or if all neurons were making less of the Nurr1 protein. When we compared +/− and +/+ Nurr1 mice, we found that the number of cells per area were similar in density but the amount of protein produced per cell was reduced. As one copy of the Nurr1 gene is knocked out in Nurr1 +/− mice, we expected an approximate 50% reduction on the amount of Nurr1 protein produced. Based on western blot and quantitative PCR on tissue from Nurr1 +/− mice, an approximately 40% reduction in Nurr1 protein and mRNA was reported (Luo et al., 2010; Rojas et al., 2010). Based on the quantified mathematical value obtained for chromogen intensity using Q-IHC, there was a 49% reduction in the amount of Nurr1 protein in the parietal/temporal cortex in +/− mice. In contrast, in the SNpc and VTA, Nurr1 protein in +/− was reduced by only 33% and 22%, respectively, when compared to the +/+ mice. These data suggest that there may be different compensatory mechanisms that can maintain Nurr1 expression in the Nurr1 +/− genotype. Based on previous research, Nurr1 mRNA expression appears to be dynamically regulated by dopamine neuron activity (Eells et al., 2012). In dopamine neurons, blocking D2 receptor increases Nurr1 expression while stimulation of dopamine receptors decreased Nurr1 expression (Eells et al., 2012). Since Nurr1 is implicated in the regulation of dopamine synthesis (i.e., tyrosine hydroxylase and GTP cyclohydrolase expression) as well as dopamine release, altered Nurr1 will likely have effects on subsequent dopamine neurotransmission. Therefore, in dopamine neurons, Nurr1 likely functions in a part of feedback loop linking neuron activity, gene expression, and dopamine neurotransmission (Sakurada et al., 1999; Hermanson et al., 2003; Jankovic et al., 2005; Eells et al., 2006; Gil et al., 2007). This mechanism could explain why the mesencephalic dopamine neurons do not show the expected 50% reduction of Nurr1 protein in +/− mice. It is currently unclear how or if Nurr1 functions in other neuron types to regulate neurotransmission.

As Nurr1 is an immediate early gene, expression of Nurr1 appears to be important for regulating its function (Chu et al., 2002; Rojas et al., 2010; Wassouf et al., 2018). Previous reports have demonstrated that Nurr1 can be induced rapidly, however, most of these studies are based on Nurr1 mRNA expression. Here we investigated Nurr1 protein expression after a brief seizure induced with PTZ. Nurr1 mRNA expression has been shown to be increased in the hippocampus following kainic acid and electroshock seizures (Xing et al., 1997; Crispino et al., 1998). Xing et al. found, following a single electroconvulsive seizure, Nurr1 mRNA increased within 15 min but was back close to base line levels by 2 h (Xing et al., 1997). Based on our IHC analysis after a PTZ seizure, we found induction of Nurr1 in the dentate gyrus but no apparent induction or intensification of expression in other areas. Using Q-IHC, we observed relatively rapid induction of Nurr1 protein expression, between 1 and 2 h (∼2 h) and a limited duration of expression, returning to base line by 8 h. This suggests a duration of Nurr1 protein expression of approximately 6 h. Due to this duration of Nurr1 protein expression, the neurons with basal Nurr1 expression likely require continual expression and this could be modified relatively quickly. Our data in the various regions in the +/− mice, suggests that this process could be altered in some populations to maintain Nurr1 expression in the +/− mice.

Because of this rapid turnover of Nurr1 protein, maintenance of Nurr1 expression appears to require environmental stimuli and pathological processes can disrupt this regulation. Alterations in Nurr1, particularly reduced Nurr1 expression, have been reported in neuropsychiatric and neurologic diseases (Bannon et al., 2002, 2004; Chu et al., 2006; Xing et al., 2006; Koo et al., 2015; Gandal et al., 2018). Within the mesencephalic dopamine neurons, Nurr1 expression is reduced with aging and in patients with Parkinson’s disease (Chu et al., 2002, 2006). Additionally, in patients with substance use disorder, specifically cocaine and opioid addiction, Nurr1 levels were found reduced in these dopamine neurons (Bannon et al., 2002, 2004; Koo et al., 2015). In tissue from Alzheimer’s disease patients, Nurr1 levels were reduced in the cortex and subiculum of the hippocampus (Moon et al., 2015). In patients with schizophrenia and bipolar disorder, a reduction in Nurr1 expression was reported in the deep layer of cortex (Xing et al., 2006). Therefore, targeting Nurr1 function could provide a therapeutic mechanism to treat these diseases. Although no endogenous ligands have been found to be necessary for Nurr1 function the way thyroid hormone and steroid hormone receptors work, several endogenous molecules and binding partners have been identified (de Vera et al., 2016). Additionally, several drugs have recently been identified with high specificity for Nurr1 binding and inducing Nurr1 target gene expression (Dong et al., 2016). The Nurr1 agonists amodiaquine, hydroxychloroquine, and SA00025 have been shown to attenuate deficits in animal models of Parkinson’s disease (Kim et al., 2015; Smith et al., 2015; Hedya et al., 2019). Amodiaquine was also effective at reducing Aβ plaques and improved cognitive function in the 5X transgenic mouse model of Alzheimer’s disease (Moon et al., 2019). Two retinoid X receptor agonists, bexarotine and IRX4204, with specificity for activating RXR-Nurr1 heterodimers, have also shown efficacy for protection from the pathology associated with Parkinson’s and Alzheimer’s disease models (McFarland et al., 2013; Wang et al., 2016; Spathis et al., 2017). These observations raise the possibility of using Nurr1 agonists to normalize Nurr1 expression and/or function to mitigate symptoms and delay or reverse the pathological process in neurological disorders associated with reduced Nurr1 expression. As the potential to use these drugs clinically becomes closer to a reality, the understanding of Nurr1 expression and function in other areas of the brain becomes more important.

## Conclusion

Using Q-IHC to measure Nurr1 protein expression revealed a wide range of expression levels across various neuronal populations. Additionally, it appears that the Nurr1 +/− genotype differentially affects Nurr1 protein expression in a region-specific way with expression in most region is considerably less than the expected 50%. Because Nurr1 expression can be induced rapidly and the protein appears to have a relatively rapid turnover of an estimated 4 h, it is important to understand the mechanisms that maintain Nurr1 expression in various neuronal types. These data are important for understanding the regional regulation of Nurr1 protein expression and how Nurr1 functions across various neuronal populations.

## Data Availability Statement

The raw data supporting the conclusions of this article will be made available by the authors, without undue reservation.

## Ethics Statement

The animal study was reviewed and approved by the Mississippi State University and East Carolina University IACUC.

## Author Contributions

JE and EK designed the experiments and wrote the manuscript. EK, SG-R, JN, and HP conducted the experimental procedures. All authors have read and approved the manuscript.

## Conflict of Interest

The authors declare that the research was conducted in the absence of any commercial or financial relationships that could be construed as a potential conflict of interest.
